# Color difference threshold of chromostereopsis induced by flat display emission

**DOI:** 10.3389/fpsyg.2015.00337

**Published:** 2015-04-02

**Authors:** Maris Ozolinsh, Kristine Muizniece

**Affiliations:** ^1^Institute of Solid State Physics, University of LatviaRiga, Latvia; ^2^Department of Optometry and Vision Science, University of LatviaRiga, Latvia

**Keywords:** chromostereopsis, eye chromatic aberrations, fusion of retinal images, stereo disparity, perception threshold

## Abstract

The study of chromostereopsis has gained attention in the backdrop of the use of computer displays in daily life. In this context, we analyze the illusory depth sense using planar color images presented on a computer screen. We determine the color difference threshold required to induce an illusory sense of depth psychometrically using a constant stimuli paradigm. Isoluminant stimuli are presented on a computer screen, which stimuli are aligned along the blue–red line in the computer display CIE xyY color space. Stereo disparity is generated by increasing the color difference between the central and surrounding areas of the stimuli with both areas consisting of random dots on a black background. The observed altering of illusory depth sense, thus also stereo disparity is validated using the “center-of-gravity” model. The induced illusory sense of the depth effect undergoes color reversal upon varying the binocular lateral eye pupil covering conditions (lateral or medial). Analysis of the retinal image point spread function for the display red and blue pixel radiation validates the altering of chromostereopsis retinal disparity achieved by increasing the color difference, and also the chromostereopsis color reversal caused by varying the eye pupil covering conditions.

## Introduction

The ability of the human eye to visualize its surrounding environment is excellent despite the fact that its optics is distinctly non-ideal. Over an extended evolution process, the human brain has adapted to the non-ideality of the human visual system. The various neural pathways of visual inputs correct our visual perception in the most economical manner. In this context, over the past few decades, life has changed relatively suddenly in terms of living conditions and habits, and many new problems have begun to affect visual perception. This is partly due to the fact that nowadays people spend long hours looking at colorful but flat visual stimuli. Thus, new considerations have to be made regarding the eye and its perception. Consequently, the study and impact of colored stimulus on the human eye via different display devices forms a primary concern.

In the above mentioned context, we first note that the optical structure of the eye possesses imperfect form factors and presents refractive index heterogeneity at different wavelengths (Gross et al., 2008; Howard and Rogers, 2012). The eye segments like other optical systems exhibit monochromatic and chromatic aberrations as well as transversal and longitudinal aberrations (Thibos et al., 1990). Chromatic aberrations result in a person’s perception of a colorful flat surface as an image consisting of several layers. Each of these layers is characterized by its inherent light wavelength or color (Thompson et al., 1993; Hong et al., 2011). The resulting effect is readily observed in many daily situations, for e.g., a subject will have the impression that a blue river or a black highway on a map appears to float slightly above the map surface instead of remaining located on the plane of the map. Further, this phenomenon manifests itself more clearly at night when the pupil diameter is increased. This phenomenon is known as (induced) color stereovision or chromostereopsis (Vos, 1960, 2008; Kishto, 1965; Ye et al., 1992; Thompson et al., 1993; Westheimer, 2008, 2013a).

Human beings also possess the ability to perceive the depth of a visual stimulus while analyzing image content This depth processing is performed by processing cues regarding the size or placement of the stimulus (Gross et al., 2008; Howard and Rogers, 2012; Dresp and Reeves, 2014). The perception of the depth (stereovision or stereopsis) occurs on the basis of the neural processing of input from both the left and right eyes.

The human brain can merge the slightly differential inputs from both eyes into one image. If the source of the inputs is flat, then stereo disparity (the horizontal difference in retinal images or image elements) can be induced by presenting slightly different images to the left and right eyes. These images can be separated optically by using anaglyphs or by use of stereo goggles while watching films on stereo projectors. Retinal stereo disparity can also occur because of light rays following different paths in the eye media, particularly when the image has a wide wavelength spectrum.

The merging of binocular retinal images has been described elsewhere for disparities originating from different kind of sources: planar or 3D, random dot or continuous, and black–white or color sources (Julesz, 1981; Simmons and Kingdom, 2002; Howard and Rogers, 2012; Ozolinsh et al., 2012). Further, induced stereopsis and stereodisparity evaluation have also recently been reported in people who spend a large percentage of time viewing computer screens (Hayashi et al., 2012).

The most commonly induced color stereovision can be observed if the stimulus contains elements of discrete spatial divisions, and further contains colors that lie far from each other on the spectrum, e.g., blue lettering on a red background. The special features of this visual effect form a primary concern in the fields of visual information design and computer displays (Vos, 2008; Chen et al., 2012).

With regard to the study of chromostereopsis, further analysis looks at longitudinal and transversal/lateral aberrations. Because of longitudinal aberrations in the eye, rays of different colors are focused at different planes in the eye, and not all these planes coincide with the retinal plane.

In the case of lateral aberrations, parallel rays of different wavelengths focus at non-corresponding retinal positions of each eye during binocular viewing. This lateral shift of focus depends on the dispersion of the eye’s refractive indices and on the angle between the incident rays and line of sight. These chromatic aberrations are observable for the case of oblique incidence. Lateral aberrations cause illusory stereo effects.

In studying chromostereopsis, many investigators have analyzed the Stiles–Crawford effect, which is the directional sensitivity of the cone photoreceptors; light entering the eye near the pupil’s periphery produces a lower photoreceptor response when compared with that produced with light of equal intensity entering near the pupil center. The Stiles–Crawford effect can be analyzed from the perspective of the origin of binocular disparity of illusory stereopsis (Ye et al., 1992; Applegate and Lakshminarayanan, 1993; Westheimer, 2013b). The Stiles–Crawford effect is considered a reason for chromostereopsis sign reversal (Winn et al., 1995).

Most studies have thus far focused on the investigation of chromostereopsis when visual stimuli consist of colors with a large span of spectral content. The aim of the present study is: (a) to investigate the binocular perception of color images and the generation of the illusory effect of stereopsis when the scene contains stimuli with small differences in the colors’ dominant wavelengths; (b) to evaluate the threshold of the minimum color difference necessary to induce chromostereopsis.

## Experimental Setup

### Stimuli

**FIGURE 1:**
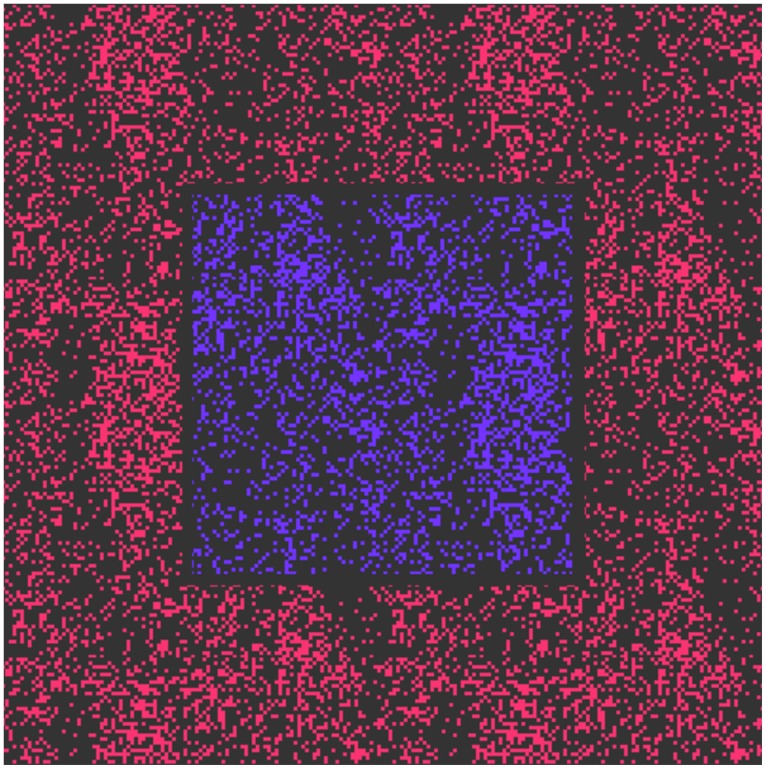
Stimulus presented on a planar light-emitting diode (LED) display. The area of the central section is 300 × 300 pixel and the viewing area is 600 × 600 pixel.

In our study on the color difference threshold of chromostereopsis induced by flat display emissions, we used visual stimuli consisting of two sections. Both these sections, which comprised central and surrounding areas (**Figure 1**), were generated as random dots. These dots formed colored elementary areas on a black background, and these elementary areas (whose color was generated by monitor luminophores) in the central area were of a slightly different color from that of the surrounding dots. The colors of these dots lay along the blue–red line of the color primaries of the display in the CIE xy diagram.

We used such visual stimuli to ensure that there were a sufficient number of vertically oriented borders in the stimulus area. We assume that this fact facilitated binocular depth sense generation. Moreover, the use of a black-colored (instead of white-colored) stimuli structure strengthened the stimulus viewing luminance, contrast, and thus the stereo disparity formation.

**FIGURE 2:**
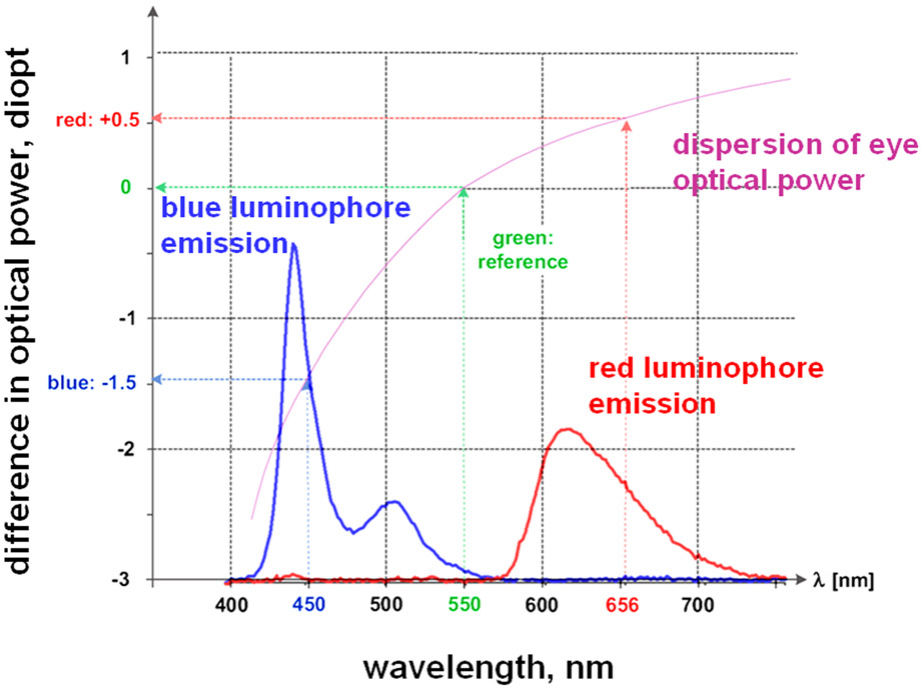
Blue and red pixel emission spectra (used on the LED screen in the trials) together with the variation in the eye’s refractive power with emission wavelength (latter data from Gross et al., 2008).

The colors of the stimuli corresponded to the magenta region, and dots were generated using two colored light sources (red and blue luminophores) on a DELL U2412M display with a pixel pitch of 0.27 mm (**Figure 2**). The observation distance was 0.8 m. Therefore, displaying 1 pixel corresponded to an angle around the visual limit of 1′. The central section of the stimulus comprised a square containing pixels of the central color, and the square size was 300 × 300 pixel. The surrounding section covered an area of 600 × 600 pixel and comprised a dot structure of another (surrounding) color. The minimum area of the elementary point dot was 3 × 3 pixel. Due to eye transversal chromatic aberrations rays emitted from one monitor pixel with different color excite photoreceptors at slightly shifted retinal positions, if the rays are not aligned along the eye achromatic axis. For the oblique rays observing the stimuli with angular size inside of 10° the maximum angular dispersion of polychromatic beam inside the eye with central wavelengths of 497 nm (blue luminophore of CRT monitor) and 605 nm (red luminophore) can reach 3 arc min (Thibos et al., 1990; Rynders et al., 1995). That is more than the size of point spread function (PSF) of the focused eye in normal vision conditions.

The average density of colored dots within the stimuli area (duty cycle) of the colored pixels was r ≈ 0.26 and the average dot area was ∼32 pixel area units. Vertical colors’ border count per one horizontal scan of the central area equaled to ≈40. The angular size of the central part was slightly greater than 5°, which is larger than the angle comprising the eye fixation direction, i.e., line of sight and the eye optical (“achromatic”) axis. The chromatic characteristics of the stimuli were measured by means of an OceanOptics 4000 Fiberoptic spectrometer and a Minolta CS-100A Luminance and Color Meter. The Minolta CS-100A measures CIE xy coordinates according to the CIE chromaticity 1931 xyY color space (the uncertainty of the device luminance measurements at the measured stimuli range was ±2%).

The saturation of the stimuli was always set at the maximum allowed by the display. The display area outside the stimulus corresponded to an angle of ≈20°, and it consisted of a neutral gray background corresponding to CIE values of x_o_ = 0.316, y_o_ = 0.336, and Y_o_ = 16.4 cd/m^2^ (this value is close to the luminance of the color dots on the monitor screen). Experiments were carried out in semi-darkness under fluorescent-lamp global and incandescent-lamp local lighting conditions. The experimental room illumination was adjusted such that the luminance of the almost neutral wall (the chromaticity CIE values of x_SR_ = 0.393 and y_SR_ = 0.379) behind the monitor coincided with the monitor background area luminance.

### Procedure

The abovementioned stimuli were presented in trials in which the central and surrounding color pair differences were varied randomly. Each trial stimuli color content was centered around the trial symmetry center, where stimuli central and peripheral dots had the same blue/red content. The difference between the stimuli colors, i.e., for the central area dots more blue vs. red (when compared with stimulus color blue/red content in the trial stimuli symmetry center) in both the central and peripheral sections were symmetrically varied while attempting to maintain the stimuli isoluminance when moving away from the trial symmetry center. Up to 30 presentations were made per trial for each color-difference pair. The multi-choice task paradigm was used to evaluate the psychophysical testing of the observers’ vision. The observers’ task was to respond to the question: “Central part or peripheral part: Which part is closer?” The central part response was assigned a value of “1” and that for the peripheral part was assigned “0,” and the responses were summed. The psychometric curves R(N) were fitted to a sigmoid function (wherein the trial sample set number N# was used as the ordinate), and the slope parameter σ was used to define the effect threshold in stimuli numbering metrics (N_Th_).

The sets of trial colored-image pairs were measured, and their colors were located in the CIE xy diagram. The threshold value N_Th_ was recalculated to determine the color difference threshold Δλ_Th_ (the central and surrounding color coordinates of the N-th sample were projected from the initiating point towards the color gamut opposite border, i.e., crossing the white point O in the CIE xy diagram).

The participants comprised two male and eight female observers. Nine subjects were school students studying optometry (age span 18–22), and one of the other participants was a 63-year-old presbyopic male observer who used the corresponding optical power goggles enabling him to see clearly red stimuli. Basic measurements were performed for “trained” observers (both the authors of the present study, a younger student who did not use viewing aids and an elderly presbyopic patient).

In order to vary the experimental conditions, we employed partial bilateral covering of the eye pupils, which produces an additional type of retinal stereo disparity. During these experiments, the observer was asked to place his head in a special mounting to avoid lateral head movements.

The study was conducted in accordance with the principles embodied in the Declaration of Helsinki Code of Ethics of the World Medical Association. All participants were unaware of the specific aims and methods of the study.

## Results

Our experiments with non-covered eye pupils allowed the determination of the minimum color difference causing chromostereopsis for stimuli created by red–blue luminophores. We attempted to ensure that the stimuli luminance was constant during each trial (as allowed by digital control of the computer’s video output). The measured stimuli color range varied within the CIE xyY coordinates of x = 0.226–0.245 and y = 0.140–0.153, and the color spot luminance varied within the mentioned color range as Y = 15.7–17.2 cd/m^2^. Adaptation to illumination and luminance levels closer to mesopic conditions can lead to a shift in the eye’s red/blue color sensitivity (position and height of eye spectral sensitivity V_λ_ curve). In this context, we remark that adaptation to the background illumination level and the individual eye spectral sensitivity of observers were not examined in our study. To stabilize the perception and to obtain data for a smooth psychophysical curve, the measurements for trained observers were obtained at least 30 min after adaptation to the experimental illumination conditions.

The filling of color spots was randomly distributed over the stimulus area. The actual luminance of the stimuli area had a standard deviation of SD_Y_ ≈ 1 cd/m^2^, and the mean luminance was Y = 4.3 cd/m^2^. The monitor white point O CIE xy chromaticity coordinates were determined as x_o_ = 0.316 and y_o_ = 0.336.

Participants experienced illusory depth perception while viewing planar color images when they were observing the stimuli binocularly. Monocular viewing did not lead to any 3D perception. Binocular stereovision, i.e., stereopsis is detectable if the color difference between the central and surrounding sections of the trial stimulus is sufficient and if the image texture is optimum with a sufficient number of vertically oriented structure transients.

We hypothesized lateral aberrations as the main cause of the illusory depth effect experienced by the observer upon viewing the color stimuli on a plane display. The perceived stereopsis and the stability of observation depend on the accommodation and fixation habits concerning the viewing of such composite images, the geometry, and decentralization of the pupil, its distraction degree, and the pupil aperture shape. The trial results of naïve patients confirmed their perception of color stereopsis; however, their observation results did not aid in building a satisfactory set of psychometric curves for threshold evaluation. The relatively low mean luminance of the stimulus area could also facilitate instability of the effect polarity (Kishto, 1965).

**FIGURE 3:**
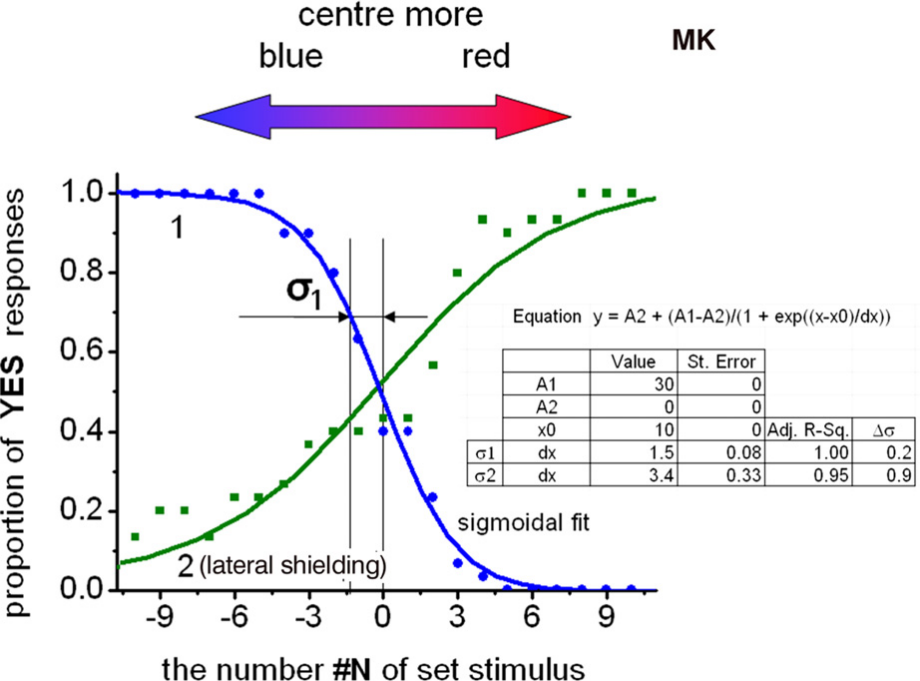
Psychometric curves to determine the threshold of color difference to detect the induced color stereo effect. The curves are – corresponding to viewing with uncovered pupils (curve 1) and curve corresponding to viewing with lateral shielding (curve 2). Parameters of sigmoidal fit of experimental points and of statistical data analysis are depicted in inset.

**Figure 3** (curve 1) shows the psychometric plot of the stable perception of color stereopsis when observing a trial set similar to the stimulus shown in **Figure 1**. Subjects were asked to concentrate only on their sense of depth and ignore any cues arising from their perception of color. The arrows at the top of figure show the direction of corresponding color increase in the central part of the image.

**FIGURE 4:**
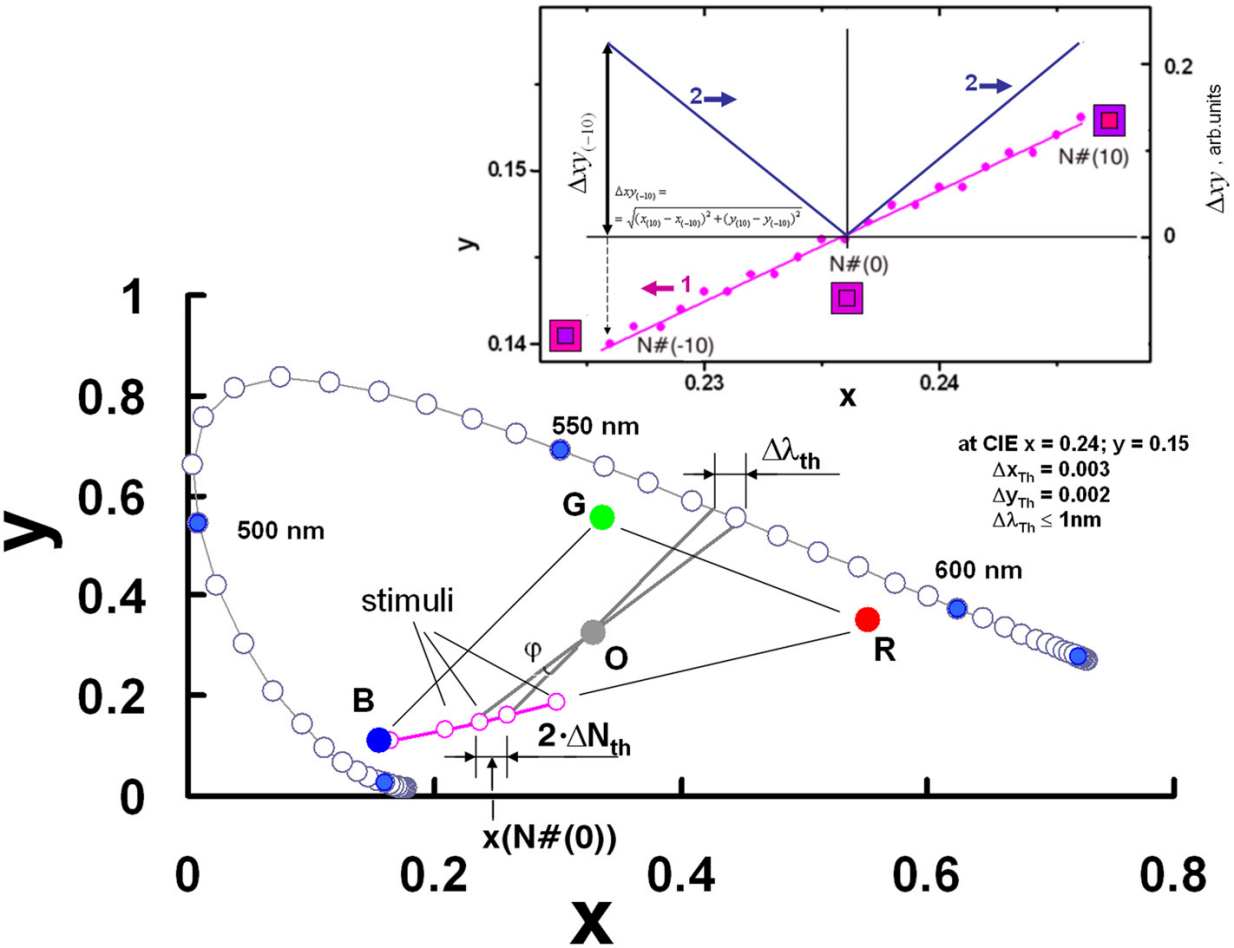
Schematical presentation of the trial stimuli in CIE xy chart – its central part chromaticity coordinates, and the principles of recalculation of the color difference threshold in the complimentary wavelength metrics. In the upper right corner the trial stimuli central-area dot chromaticity CIE xy coordinates are depicted – curve 1, and varying of the color difference between stimuli central and peripheral parts xy for trial stimuli N(10) to N(10) – curve 2, shown.

Next, we prepared 21 trial stimuli [from N#(-10) to N#(10)] that exhibited a gradual increase in the central redness. In a synchronous manner, the increase of redness (see the inset of **Figure 4**) was offset by a decrease of bluishness in the central part, and with the opposite varying of colors in the peripheral part of the stimuli. The surrounding and central areas of the stimuli N#(0) consisted of randomly distributed active elements emitting the same color. In addition, the ratio of the blue to red of the stimuli color was opposite for stimuli with positive vs. negative indexing, for e.g., for N#(-5) and N#(5), in the left and the right sides of the central stimuli N#(0) in **Figure 3**.

Thus, each trial had stimuli with 10 difference values between the colored dots of the central and peripheral parts. The resulting psychophysical curves allowed the determination of the threshold of the color difference. The inset of **Figure 4** schematically depicts the stimuli chromatic x coordinates (curve 1) for the central part and the variation in the color difference $\Delta xy=\sqrt{{\left(x_{c}-x_{p}\right)}^{2}+{\left(y_{c}-y_{p}\right)}^{2}}$ (wherein the subscripts indicate the indexing points: central or peripheral) between the central and peripheral parts in CIE xy metrics (curve 2) along the trial stimuli set.

Another key point of our study involved the presentation of the results in terms of reasonable metrics for color research. As the color of the stimuli lay on the line connecting the display’s blue and red luminophore CIE xy chart coordinates, we proposed also the stimuli’s complementary color dominant wavelength as a potential measure of the stimuli (schematically shown in **Figure 4**).

The colorimetric coordinates in the CIE xy chart of the following points were first determined. First, we determined x_i_ and y_i_ for the set of trial stimuli on both sides of the trial equilibrium point N#(0). For equilibrium point N#(0) the central and surrounding colors are identical with coordinates corresponding to – x_0_ and y_0_. Secondly, we determined the virtual points N#(-σ) and N#(+σ) with coordinates x_0-σ_, y_0-σ_ and x_0+σ_, y_0+σ_. These points were shifted along the monitor’s blue–red line in the CIE xy chart from both sides of the equilibrium point N#(0) at the distance that corresponds to the threshold distance σ in the metrics of stimuli numbering. The values of x_0-σ_, y_0-σ_ and x_0+σ_, y_0+σ_ were determined by performing a linear interpolation of data along the stimuli line in corresponding CIE xy chart area. The triangle with vertices of the O-point, x_0-σ_, y_0-σ_, and x_0+σ_, y_0+σ_ determined the angle of the O-point vertex value φ (**Figure 4**). The projection of this angle on the color locus green–yellow area allowed the use of CIE complementary wavelength metrics for the stimuli color difference threshold. The data in **Figure 3** can be used to determine the color stereopsis threshold of N_Th_ = σ_1_ = 1.5 for the uncovered pupil conditions at the color equilibrium point specified by x = 0.24, y = 0.15 in the CIE xy chart. Average luminance of the display screen in these trials was Y = 4.3 cd/m^2^. Statistical analysis yielded the uncertainty of the sigmoid function data in **Figure 3** – Δσ = ± 0.2 for *p* = 0.95. The average CIE chart parameter thresholds for chromostereopsis on the display’s red–blue line of the group for two trained observers were Δx_Th_ = 0.003 and Δy_Th_ = 0.004. The data for the older presbyopic observer’s color stereopsis when compared with that of the younger observer revealed higher data scattering, however, the sigmoid fit yielded σ values that were not significantly larger.

Our recalculations according to the abovementioned algorithm yielded the threshold value for the uncovered pupil color stereopsis as φ_Th_ = 0.7°, and the corresponding threshold in terms of the complementary wavelength metrics was Δλ_Th_ ≤ 1 nm. We note that the last set of metrics can be universally applied to trichromatic stimuli along other axes in the CIE xy diagram, and for stimuli along the blue–red display line, the metric set can be very resolvable.

**Figure 3** (curve 2) also shows the psychometric plot under conditions wherein the eye pupil areas were partially covered. The observers in this case used a trial frame with semi-circle apertures, which allowed centering of the apertures and the realizing of synchronous horizontal flips of the covered pupil area for the left and right eyes. Variation in the covered pupil areas (medial or lateral) switched the stereopsis polarity. Curve 2 shows the psychometric curve slope and the slope parameters (**Figure 3**) for observation conditions with lateral shielding.

## Discussion

The angular size of the central area in our study was slightly larger than the eye angles between the fixation and optical axes (Thibos et al., 1990). In the experiments, we asked observers to make their decision about the perceived mutual displacement of a larger area that lay outside the range comprising these visual axes, including the direction along the eye’s “achromatic” axis.

The light rays emitted from the stimuli area are subject to chromatic aberrations in the eye. Furthermore, binocular viewing leads to disparity based on the color content of the stimuli, and the summary effect should be considered taking into account integration over all viewing angles. When permission was given to the naïve observers to freely move the eye fixation line over the entire central area, the “noise” of their decisions increased. This type of instability in decision-making and increase in the noise can be due to various reasons, all of which are eventually due to the large viewing angle of all stimuli.

The results for the observers can be grouped in the following manner. Subjects in the first group experience stereopsis (the stimuli center is in front of stimuli periphery or vice versa) in the range of demonstrated trial stimuli. However, the observations are not stable; i.e., we can state “stereopsis is present; however, it does not have a measureable threshold,” which was true for eight naïve observers. As regards the second observation group, the subjects experience stable stereopsis; however, their responses are not clearly distinct. The shape and width of the fitted psychometric curve are definite, but the data points are noisy, and they do not lie along the fitting curve. This result was valid for two trained observers. In the third observation group, subjects are well concentrated for such experiments and they experience stable stereopsis. In this case, the psychometric curves are less noisy and the threshold can be clearly detected for the same two trained observers as measured in another test series with the same parameters. Actually, all participants including the naïve observers experienced the depth effect in the range of demonstrated trial stimuli, however, the responses of the naïve subjects were not stable. We did not statistically consider the oscillating responses in evaluating the color stereopsis for the group of naïve observers. The impossibility of obtaining consistent psychophysical curves for these observers with non-stable stereopsis prevents us from combining their data without violating the description (including mathematical description) of the effect.

Previous reports (Rozkova et al., 2012) have indicated eye strabismus as a possible cause that can alter positive (“Red-in-front”) color stereopsis to negative (“Blue-in-front”) color stereopsis. Binocular features such as eye tropias and phorias, fixation disparity, and the possibility of fixating on an imaginary stereo plane can all influence the color stereopsis sign and observation stability.

In previous studies, the stimuli mostly were monochromatic (Ye et al., 1991; Thompson et al., 1993) or generated on a computer screen by display color primaries (Simmons and Kingdom, 2002; Kitaoka et al., 2006; Hayashi et al., 2012). In our study, we used a series of magenta stimuli around the “equilibrium point” (wherein the colors in stimuli’s central and surrounding areas have equal chromaticity coordinates). Each of the stimuli dots sends emission from the blue and red luminophores toward the eye retinal plane at two distinct retina areas: one for blue pixel and other for red one. These areas coincide only if the emission rays pass along the eye achromatic axis. Due to longitudinal chromatic aberrations of the eye, these projections are not well focused at the retinal plane. We use the term “center-of-gravity,” which was introduced by Kitaoka et al. (2006) to characterize this situation.

For the stimulus at the trial equilibrium point, when center and peripheral dots are of the same color, each of the stimulus dots of both the center and surrounding areas projects an identical spatial distribution of the chromatic emission on the eye’s retinal plane. The centers-of-gravity for both primary and secondary color contributions are situated in the same retinal position. No stereo disparity can be generated in this case. When colors of central and surrounding areas move away from the trial equilibrium point in opposite directions, the central and surrounding dots due to their dissimilar color project different spatial distribution of emission on the retina. This difference creates stereo disparity of the illusory depth sense via binocular observation in such cases, when eyes’ pupils are not circular, for e.g., semicircular with opposite geometry regarding to the face symmetry.

**FIGURE 5:**
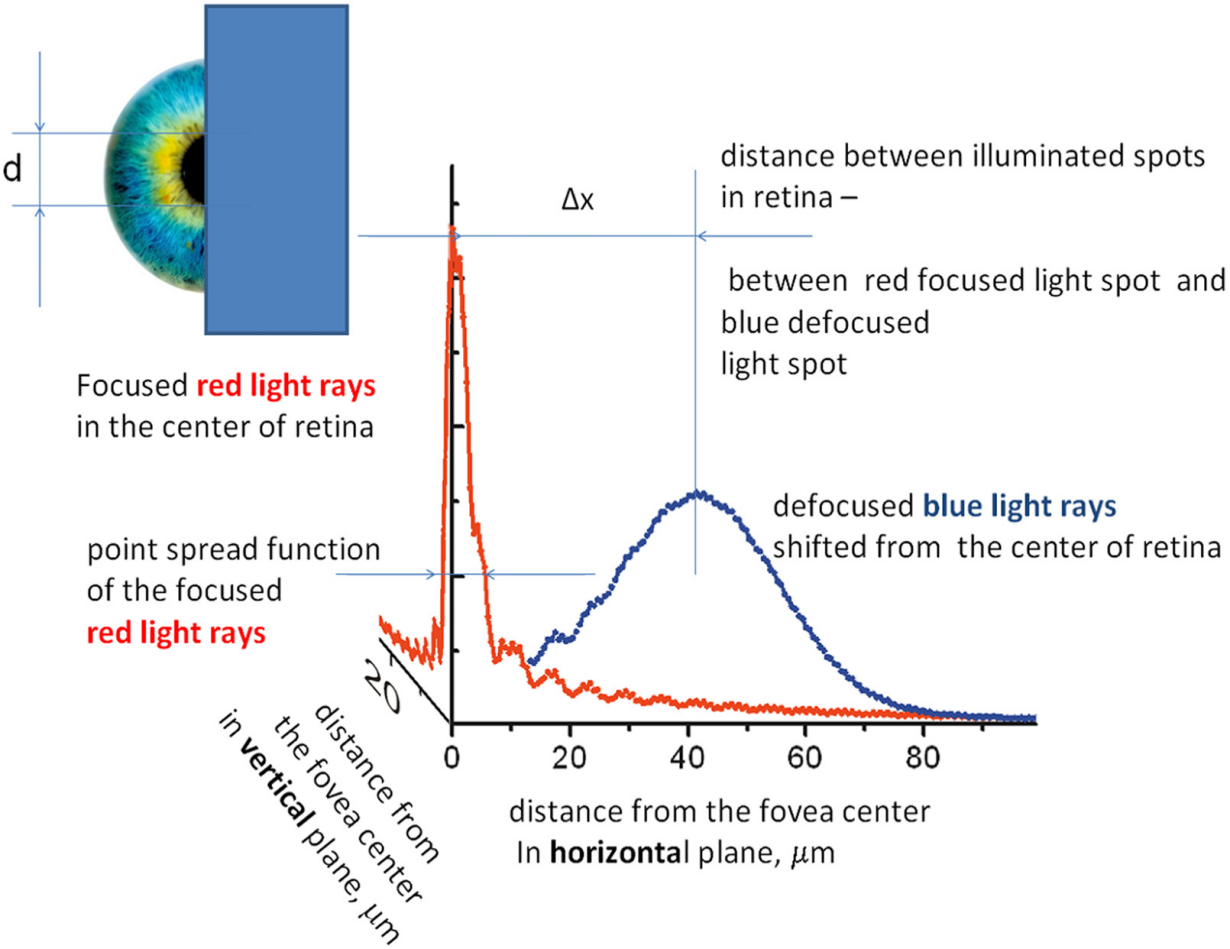
Retinal illumination at the eye’s horizontal and vertical planes from a single-display R + B pixel calculated from the point spread function (PSF) and its defocusing for blue emission. Calculations are performed for eye with half the pupil covered. Inset shows the covering of the eye pupils.

The center-of-gravity model can be used to explain the results (**Figure 3**, curve 2) of studies on chromostereopsis when observations are made through shielded eye pupils. We used the Fourier transformation of the aperture for radiation through the eye pupil of the half-covered eye for red emission, and we evaluated the circle of confusion for non-focused and wavelength-diffused blue emission to subsequently calculate the retinal illumination. **Figure 5** shows the determined PSF of the half-covered eye at the focus point for the displayed red emission and defocused illumination for blue emission (the color caused an optical power shift of ΔP ≈ 1.3 in the eye, according to **Figure 2**).

The medial or lateral covering of pupils distinctly switches the polarity of the induced depth sense, i.e., the blue image plane is perceived in front of the red image plane and vice versa. The right and left eyes exhibit mirror symmetry of retinal illumination, and further, non-oblique rays (due to their lateral aberrations) can contribute to the induced stereo disparity. In addition, the effect polarity or positive vs. negative switching, i.e., the perception of blue being closer than red and vice versa, occurs upon altering the geometry of eye pupil covering. Our observations confirm this phenomenon, i.e., the pronounced switching of the depth effect polarity by altering the shielding of half of the pupils from medial to lateral. However, the experimental points on the psychophysical curve in the case of shielding exhibited a larger deviation at first, and the color difference threshold at least doubled subsequently. Such variance can perhaps be explained by the minimization and decentralization of the pupil area and decrease in retinal illumination.

## Conclusion

We studied chromostereopsis by continuously increasing the color difference of the central area and the surrounding dots. The simultaneous increase in stereo disparity was substantiated by application of the center-of-gravity model.

We determined that chromostereopsis becomes detectable for the color difference of image elements above the threshold with values of Δx_Th_ = 0.003 and Δy_Th_ = 0.004 according to CIE chromaticity coordinates that can be transposed to stimuli complimentary wavelength metrics as 1 nm. Such color difference threshold parameters are valid for the case of red–blue computer display radiation determined at the color difference equilibrium point of x = 0.24, y = 0.15 in the CIE xy chart for stimuli with an averaged luminance of Y = 4.3 cd/m^2^. These values were determined under viewing conditions in which no eye-pupil-shielding obstacles were used. Symmetrical bilateral medial or lateral screening of both eye pupils led to reversal of the illusory effect, i.e., from the perception of blue in front of red to that of red in front of blue. Our findings are important in terms of evaluating induced stereopsis effects on images produced on planar screens when the digital chromatic output consists of more or less saturated short- and long-wavelength colors. The use of colors with a large wavelength span can cause an essential distortion of visual information.
